# Long-term prognosis in patients continuing taking antithrombotics after peptic ulcer bleeding

**DOI:** 10.3748/wjg.v23.i4.723

**Published:** 2017-01-28

**Authors:** Xi-Xu Wang, Bo Dong, Biao Hong, Yi-Qun Gong, Wei Wang, Jue Wang, Zhen-Yu Zhou, Wei-Jun Jiang

**Affiliations:** Xi-Xu Wang, Bo Dong, Biao Hong, Yi-Qun Gong, Wei Wang, Jue Wang, Department of Vascular Surgery, Shanghai Tongren Hospital, Shanghai 200336, China; Zhen-Yu Zhou, Wei-Jun Jiang, Department of Gastroenterology, Shanghai Tongren Hospital, Shanghai 200336, China

**Keywords:** Peptic ulcer bleeding, Antithrombotics, Cardiovascular disease, Risk factor, Survival curve

## Abstract

**AIM:**

To investigate the long-term prognosis in peptic ulcer patients continuing taking antithrombotics after ulcer bleeding, and to determine the risk factors that influence the prognosis.

**METHODS:**

All clinical data of peptic ulcer patients treated from January 1, 2009 to January 1, 2014 were retrospectively collected and analyzed. Patients were divided into either a continuing group to continue taking antithrombotic drugs after ulcer bleeding or a discontinuing group to discontinue antithrombotic drugs. The primary outcome of follow-up in peptic ulcer bleeding patients was recurrent bleeding, and secondary outcome was death or acute cardiovascular disease occurrence. The final date of follow-up was December 31, 2014. Basic demographic data, complications, and disease classifications were analyzed and compared by *t*- or χ^2^-test. The number of patients that achieved various outcomes was counted and analyzed statistically. A survival curve was drawn using the Kaplan-Meier method, and the difference was compared using the log-rank test. COX regression multivariate analysis was applied to analyze risk factors for the prognosis of peptic ulcer patients.

**RESULTS:**

A total of 167 patients were enrolled into this study. As for the baseline information, differences in age, smoking, alcohol abuse, and acute cardiovascular diseases were statistically significant between the continuing and discontinuing groups (70.8 ± 11.4 *vs* 62.4 ± 12.0, *P* < 0.001; 8 (8.2%) *vs* 15 (21.7%), *P* < 0.05; 65 (66.3%) *vs* 13 (18.8%), *P* < 0.001). At the end of the study, 18 patients had recurrent bleeding and three patients died or had acute cardiovascular disease in the continuing group, while four patients had recurrent bleeding and 15 patients died or had acute cardiovascular disease in the discontinuing group. The differences in these results were statistically significant (*P* = 0.022, *P* = 0.000). The Kaplan-Meier survival curve indicated that the incidence of recurrent bleeding was higher in patients in the continuing group, and the risk of death and developing acute cardiovascular disease was higher in patients in the discontinuing group (log-rank test, *P* = 0.000 for both). Furthermore, COX regression multivariate analysis revealed that the hazard ratio (HR) for recurrent bleeding was 2.986 (95%CI: 067-8.356, *P* = 0.015) in the continuing group, while HR for death or acute cardiovascular disease was 5.216 (95%CI: 1.035-26.278, *P* = 0.028).

**CONCLUSION:**

After the occurrence of peptic ulcer bleeding, continuing antithrombotics increases the risk of recurrent bleeding events, while discontinuing antithrombotics would increase the risk of death and developing cardiovascular disease. This suggests that clinicians should comprehensively consider the use of antithrombotics after peptic ulcer bleeding.

**Core tip:** Patients with peptic ulcer bleeding were enrolled into our study, and clinical information was analyzed by statistical method. We found that continuing antithrombotic drugs for peptic ulcer patients increased the risk of recurrent bleeding events, and discontinuing antithrombotic drugs increased the risk of death or cardiovascular events. Our results indicate that clinicians should balance the usage of antithrombotics to reduce the risk of peptic ulcer bleeding.

## INTRODUCTION

Peptic ulcer is a highly prevalent illness[1], and it tremendously threatens the health of humans due to high morbidity and severe complications[2-5]. Among all complications, peptic ulcer bleeding is one of the common clinical diseases[6]. In recent years, despite the application of proton pump inhibitors (PPIs)[7-9] and *Helicobacter pylori* eradication[10-12], the morbidity of peptic ulcer bleeding has not decreased[13] at least partially due to the use of antiplatelet agents, anticoagulants, and thrombin inhibitors. These drugs have recently been used extensively for the treatment of thromboembolic disease[14-16]. It has also been estimated that the usage of these drugs has been increasing worldwide as cardiovascular morbidity increases in the aged population[17,18]. This would induce a high incidence of peptic ulcer bleeding[19,20]. Aspirin is an antithrombotic drug that has been widely applied in view of the benefit in preventing cardiovascular disease[21,22]. However, patients with cardiovascular disease are recommended to immediately discontinue the usage of aspirin after successful endoscopic treatment of peptic ulcer bleeding, in order to prevent death or acute disease occurrence, according to the Medication Guide[23]. In a randomized double-blind study, Sung et al[24] found that recurrent bleeding events due to the continued usage of aspirin severely influences the prognosis of patients. Therefore, there is a dilemma to the clinical usage of antithrombotic drugs. Furthermore, there are few studies on antithrombotics usage for treating peptic ulcer bleeding patients, and there is increasing concern on these patients. Hence, we collected the clinical data of patients with peptic ulcer bleeding treated at our hospital in recent five years, aiming to investigate the effect of continued administration of antithrombotic drugs and identify the risk factors for prognosis.

## MATERIALS AND METHODS

### Study objects

**Figure 1:**
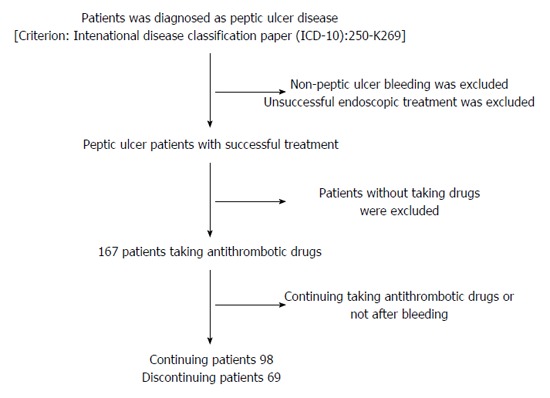
Flow of enrollment of patients with peptic ulcer.

Patients with peptic ulcer treated at Tongren Hospital affiliated to Shanghai Jiao Tong University from January 1, 2009 to January 1, 2014 were included in this study. The study ended on December 31, 2014. Upper gastrointestinal hemorrhage was defined as hemoptysis, hematochezia, melena, fainting, or dizziness with anemia. The following patients were excluded: patients with non-peptic ulcer bleeding, esophageal varices, vascular dysplasia, esophageal or gastric cancer, and ulcer perforation; patients with peptic ulcer bleeding who had an unsuccessful endoscopic treatment; patients who did not receive antithrombotic drugs after a successful therapy; patients who received PIPs to prevent damage from antithrombotic drugs. The enrollment process is shown in Figure 1.

Finally, a total of 167 patients were enrolled in this study. Based on whether drug administration was continued or discontinued after peptic ulcer bleeding healed following endoscopic treatment, the patients were divided into either a continuing group (*n* = 98) or a discontinuing group (*n* = 69). The continuing group included the patients who continued taking the drugs after the bleeding healed, while the discontinuing group included the patients who discontinued taking the drugs after the bleeding healed. All patients in this study provided a signed informed consent form, and this study was approved by the hospital ethics committee.

### Study methods

The clinical data of the patients, including demographic data and complications, were analyzed statistically. The time to end point was strictly calculated. Primary end point was recurrent bleeding events within 30 d, including hemoptysis, melaena, > 2 g/dL of hemoglobin within 24 h, and unstable blood flow (systolic blood pressure ≤ 90 mmHg or heart rate ≥ 110 times/min). Patients with one of the aforementioned or combined characteristics were defined to achieve the primary end point. Secondary outcomes were death, acute cardiovascular disease, acute myocardial infarction, and ischemia or transient ischemia. The number of patients with different outcomes was counted, and the difference was compared statistically. Survival time was collected to draw the survival rate.

### Statistical analysis

All data were analyzed using SPSS 19.0 software. Age, hemoglobin levels, and body mass index (BMI) measurements are expressed as mean ± SD. Categorical data are expressed as percentages. Measurement data following a normal distribution were compared by the *t-*test. Frequency data were compared by the χ^2^-test. Kaplan-Meier method was applied to calculate the survival rate and draw the survival curve. Differences were compared using the log-rank test. The multivariable COX proportional regression model was applied to analyze the risk factors for prognosis in patients with peptic ulcer bleeding. *P* < 0.05 was considered statistically significant.

## RESULTS

### Baseline data of patients with peptic ulcer bleeding

After screening, a total of 167 patients with peptic ulcer bleeding were enrolled into this study. Among these patients, 98 continued receiving antithrombotic drugs, and 69 discontinued. The average age of the patients who continued and discontinued receiving antithrombotic drugs was 70.8 ± 11.4 years and 62.4 ± 12.0 years, respectively (*P* = 0.000). The percentage of patients with a history of smoking or alcohol abuse was significantly higher in the continuing group than in the discontinuing group (*P* = 0.012). Furthermore, the rate of cardiovascular complications was significantly higher in the continuing group (*P* = 0.000; Table 1).

**Table 1:** Baseline information between the continuing and discontinuing groups *n* (%)

**Characteristic**	**Continuing group**	**Discontinuing group**	***T*/**χ**^2^**	***P* value**
No. of patients	98	69		
Demographic data				
Gender (male/%)	68 (69.39)	42 (60.87)	1.307	0.253
Age (mean ± SD)	70.8 ± 11.4	62.4 ± 12.0	4.588	0.000
BMI (kg/m^2^, mean ± SD)	21.4 ± 4.5	22.0 ± 4.2	0.872	0.385
Complications				
Smoking and alcohol abuse	8 (8.2)	15 (21.7)	6.284	0.012
Diabetes	32 (32.7)	15 (21.7)	2.385	0.123
Hypertension	71 (72.4)	56 (81.2)	1.338	0.247
Chronic kidney disease	23 (23.4)	8 (11.6)	3.777	0.052
Chronic obstructive pulmonary disease	10 (10.2)	8 (11.6)	0.081	0.775
Acute cardiovascular disease	65 (66.3)	13 (18.8)	36.681	0.000
Non-antithrombotic drug usage				
Aspirin	84 (85.7)	59 (85.5)	0.001	0.970
Forrest classification				
I-II	31 (31.6)	28 (40.6)	1.419	0.234
III	67 (68.4)	41 (59.4)	1.419	0.234
Hemoglobin (g/dL)	9.0 ± 2.4	8.6 ± 2.1	1.116	0.266

### Comparison of various outcomes between the two groups

Recurrent bleeding occurred in 18 patients in the continuing group and in four patients in the discontinuing group. Death or acute cardiovascular disease occurred in three patients in the continuing group and in 15 patients in the discontinuing group. The differences in the rates of primary and secondary outcomes between the two groups were statistically significant (*P* = 0.018, *P* = 0.000; Table 2).

**Table 2:** Comparison of various outcomes achieved between the two groups *n* (%)

	**Continuing group**	**Discontinuing group**	**χ^2^**	***P* value**
Recurrent ulcer bleeding events	18 (18.4)	4 (5.8)	5.594	0.018
Death or cardiovascular disease	3 (3.1)	15 (21.7)	14.689	0.000

### Survival curve in the two groups

**Figure 2:**
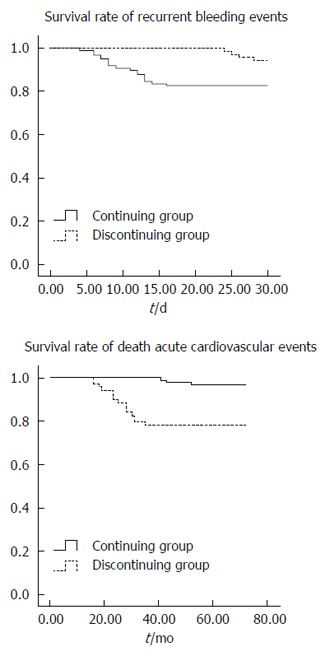
Kaplan-Meier survival curves for various outcomes.

Kaplan-Meier results indicated that bleeding occurred more frequently in patients in the continuing group, while survival rate was significantly higher in patients in the discontinuing group (Log-rank test, *P* = 0.022, *P* = 0.000; Figure 2).

### Risk factors for prognosis of patients

The multivariable COX proportional regression model indicated that continuing intake of antithrombotic drugs increased the risk of recurrent bleeding events (95%CI: 1.067-8.356, OR = 2.986, *P* = 0.015), while discontinuing intake of antithrombotic drugs increased the risk of death or acute cardiovascular disease (95%CI: 1.035-26.278, OR = 5.216, *P* = 0.028; Table 3).

**Table 3:** COX regression multivariate analysis of risk factors for prognosis

	**Recurrent ulcer bleeding events**				**Death or cardiovascular occurrence**			
	**β**	**OR**	**95%CI**	***P* value**	**β**	**OR**	**95%CI**	***P* value**
Usage of antithrombotics	1.094	2.986	1.067-8.356	0.015	1.652	5.216	1.035-26.278	0.028

Usage of antithrombotics

## DISCUSSION

Peptic ulcer is one of the most common clinical gastrointestinal tract diseases at present[6,25], and it is generally induced by damage of the gastric or duodenal mucosa. Gastric acid and protease play a crucial role in disease progression[26,27]. The aged population accounts for most of the cases, and ulcer bleeding, perforation, and pyloric obstruction are the most common complications[28-31]. *Helicobacter pylori* infection, excessive secretion of gastric acid, and excessive antithrombotic drug intake often trigger the occurrence of ulcer bleeding[32-34]. In recent years, peptic ulcer morbidity increased slowly due to medical technology progression[35]; however, the incidence of ulcer bleeding has been continuously increasing[36,37].

Cardiovascular disease is defined as ischemic or hemorrhagic disease occurring in all tissues due to atherosclerosis and blood viscosity[38]. In view of its high morbidity and mortality, more and more people, even healthy people, tend to take antithrombotics to prevent and reduce its risk[39]. However, excessive drug usage will increase the risk of bleeding in patients with ulcer bleeding.

As the aged population has an increased necessity for preventing acute cardiovascular disease, aspirin and other antithrombotics continue to be broadly used[40-42]. It has been reported that most patients with established cardiovascular disease ignore the risk of aspirin, and continue to take aspirin or other antithrombotics for secondary prevention[43,44]. Even more, the literature has shown that cardiovascular disease complications occur more frequently in patients who have discontinued antiplatelet drug therapy, compared to patients who continue this therapy[45]. Nevertheless, continuing intake of aspirin or antithrombotic drugs will increase the risk of hemorrhage complications in surgery instead[46,47]. Accordingly, clinicians could not balance the risk of cardiovascular disease and hemorrhage complications. In addition, there are few studies on the prognosis of patients with peptic ulcer bleeding. Hence, the clinical data of patients with peptic ulcer bleeding treated at our hospital were collected and analyzed to discuss the prognosis of patients, hoping to provide guidance for clinical applications.

### Age, smoking, and alcohol abuse influence the usage of antithrombotics in patients with peptic ulcer bleeding

Our study revealed that patients were older in the continuing group than in the discontinuing group, indicating that aged patients need more antithrombotics. This is consistent to the current social situation. The number of patients with cardiovascular complications was higher in the continuing group than in the discontinuing group, which is in agreement with a previous report that patients with an established disease tend to continue taking drugs. In addition, there was a difference in smoking and alcohol abuse between the two groups; there were more of these patients in the discontinuing group. It is plausible that patients taking drugs tended to reduce smoking or alcohol consumption.

### Taking antithrombotics affects survival rate in patients with peptic ulcer bleeding

Our study indicated that patients who continued to take the drugs had a higher risk of recurrent bleeding events. In contrast, patients in the discontinuing group had a higher risk of death or acute cardiovascular disease. This result is inconsistent with recent studies reporting that there was no difference between the two groups. On one hand, our follow-up time did not have a limit. However, patients with less than two months of follow-up were excluded in the study conducted by Kim et al[48]. Furthermore, it has been widely accepted that recurrent bleeding time was shorter than normal bleeding time. On the other hand, the number of patients in these two studies is different. Consistent with our results, Sung et al[24] estimated a higher incidence of recurrent bleeding events in patients continuing taking aspirin in a randomized double-blind study (a likelihood ratio of nearly 2). Through retrospective research, we also obtained similar results (a likelihood ratio of nearly 3)[49], which further supports this view. This implies that more attention should be given when continuing taking antithrombotics.

### Limitations and prospects

This study has some limitations. First, a limited number of patients could not sufficiently support our conclusion. Second, we did not distinguish different antithrombotic drugs. However, many studies have shown that the single or combined application of drugs would result in an obvious difference. Finally, the definite time of bleeding was lacking. Hence, we were not able to obtain the precise time when to discontinue or continue drugs.

In conclusion, our results demonstrate that after the occurrence of peptic ulcer bleeding, continuing the intake of drugs would increase the risk of recurrent bleeding events, while discontinuing the intake of drugs will increase the risk of death and acute cardiovascular occurrence. These indicate that clinicians must extensively weigh the benefits and risks when using antithromboticsin for treating patients with peptic ulcer bleeding.

## COMMENTS

### Background

Peptic ulcer is one of the most common gastrointestinal diseases which is generally induced by damage of the gastric or duodenal mucosa. Gastric acid and protease play a crucial role in disease progression. The aged population accounts for most of the cases, and ulcer bleeding, perforation, and pyloric obstruction are the most common complications. *Helicobacter pylori* infection, excessive secretion of gastric acid, and excessive antithrombotic drug taking would trigger the occurrence of ulcer bleeding. In recent years, peptic ulcer morbidity has increased slowly duo to medical technology progression, but ulcer bleeding incidence rate has been increasing all the time. Cardiovascular disease is defined as ischemic or hemorrhagic disease occurring in all tissues due to atherosclerosis and blood viscosity. In view of its high morbidity and mortality, more and more people, even healthy people, tend to take antithrombotics to prevent and reduce its risk. However, excessive drug usage will increase bleeding risk instead in ulcer bleeding patients.

### Research frontiers

As the aged population has an increased necessity for preventing acute cardiovascular disease, aspirin and other antithrombotics are broadly used. It is reported that most patients with established cardiovascular disease ignore the risk of aspirin and still insist in taking aspirin or other antithrombotics for secondary prevention. Even more, the literature shows that patients have cardiovascular disease complication more easily in those discontinuing antiplatelet drug therapy compared to those continuing antiplatelet drug therapy. Nevertheless, continuing aspirin or antithrombotic drugs will increase hemorrhage complication risk in surgery instead. Accordingly, clinicians could not balance risk of cardiovascular disease and hemorrhage complication.

### Innovations and breakthroughs

The authors investigated the prognosis and risk factors in peptic ulcer bleeding patients. Even more, they showed two survival curves with disparate outcomes to demonstrate survival difference in patients continuing or discontinuing taking antithrombotics. These results suggest that clinicians must take more attention in the usage of antithrombotic drugs.

### Applications

This study demonstrates that after occurrence of peptic ulcer bleeding, continuing taking drugs will increase the risk of recurrent bleeding events, and discontinuing drugs will increase risk of death and acute cardiovascular occurrence, which indicates that clinicians must weigh the risks and benefits when using antithrombotics to treat ulcer bleeding patients.

### Peer-review

Patients with ulcer peptic bleeding were enrolled in this study and clinical information was analyzed by statistical method. Authors found that continuing antithrombotic drugs in ulcer peptic patients increased the risk of recurrent bleeding events while discontinuing drugs increased risk of death or cardiovascular events. The results indicated that clinicians should balance the usage of antithrombotics to reduce risk in peptic ulcer bleeding patients
